# Beamforming and Power Control in Sensor Arrays Using Reinforcement Learning

**DOI:** 10.3390/s150306668

**Published:** 2015-03-19

**Authors:** Náthalee C. Almeida, Marcelo A.C. Fernandes, Adrião D.D. Neto

**Affiliations:** 1 UFERSA—Federal Rural University of the Semi-Árido, Pau dos Ferros 59900-000, Brazil; 2 DCA-CT-UFRN, Federal University of Rio Grande do Norte, Natal 59072-970, Brazil; E-Mails: mfernandes@dca.ufrn.br (M.A.C.F.); adriao@dca.ufrn.br (A.D.D.N.)

**Keywords:** beamforming, power control, sensor arrays, Q-learning

## Abstract

The use of beamforming and power control, combined or separately, has advantages and disadvantages, depending on the application. The combined use of beamforming and power control has been shown to be highly effective in applications involving the suppression of interference signals from different sources. However, it is necessary to identify efficient methodologies for the combined operation of these two techniques. The most appropriate technique may be obtained by means of the implementation of an intelligent agent capable of making the best selection between beamforming and power control. The present paper proposes an algorithm using reinforcement learning (RL) to determine the optimal combination of beamforming and power control in sensor arrays. The RL algorithm used was Q-learning, employing an ε-greedy policy, and training was performed using the offline method. The simulations showed that RL was effective for implementation of a switching policy involving the different techniques, taking advantage of the positive characteristics of each technique in terms of signal reception.

## 1. Introduction

Sensor arrays have been widely used in a variety of applications including estimation of the direction of arrival (DOA) of signals [1,2], tracking systems, location of sources [3], and suppression of interference signals [4], amongst others. Adaptive array systems are able to locate and track signals (of users and interferences), dynamically adjusting the sensor alignment to maximize reception, and minimizing interference using signal-processing algorithms [5]. In applications where the aim is to suppress interference signals associated with various different sources, adaptive sensor arrays can be used together with power control techniques. The development of adaptive sensor arrays using beamforming together with power control can be used to achieve better system performance, with lower consumption of energy for transmission [6,7,8,9,10,11,12,13,14,15,16,17].

Methods used to resolve the problem of combining beamforming with power control have been described [6,7], where an algorithm has been proposed that is capable of beamforming in the uplink channel, followed by the adjustment of power in this channel and in the downlink channel. In this case, the weights for the downlink channel are considered the same as those for the uplink channel. This algorithm improves the performance of the system using a Signal to Interference plus Noise Ratio (SINR).

In [8], a duality constrained least-mean-square (DCLMS) algorithm was proposed that utilizes LMS to find the optimum beamforming weights, while at same time controlling the power in both the uplink and downlink channels. A reference signal is used for beamforming, avoiding the use of additional algorithms for computation of the arrival directions of the signals. Since it is based on LMS, the algorithm presents low computational complexity, and the adaptation step for control of convergence can be selected empirically in order to better address the objectives of application of the algorithm. Updating of the transmission power only occurs after convergence of the beamforming process, taking account of successive tests of convergence and loss of performance in non-stationary environments. 

A similar algorithm is presented in [9], but with proportionality between the uplink and downlink weights. In other work [10], minimization of the transmission power in the channels is performed for each antenna individually. All these approaches assume a priori knowledge of the channel, so that it is possible to calculate the reception SINR, which then enables calculations for updating of the transmission powers in the uplink and downlink channels.

The work described in [11] presents a joint optimization of beamforming and power control in a coordinated multicell downlink system which attends multiple users per cell to maximize the minimum weighted signal-to-interference-plus-noise ratio. The optimal solution and distributed algorithm with a fast convergence rate are obtained using the nonlinear Perron-Frobenius theory and the multicell network duality. Despite operating in a distributed manner, the iterative algorithm requires instantaneous power update in a coordinated cluster by means of backhaul.

In [12], an algorithm is proposed that explores techniques of beamforming at the source and destination nodes, together with control of transmission power, in order to minimize the total transmission power of the source so that a minimum SINR threshold can be maintained in each receiver. This objective is achieved using an iterative algorithm that combines these techniques.

The work described in [13] proposes an algorithm for power control together with beamforming in the receiver, with multiple adaptive base stations, for communication in the uplink channel. An iterative optimization algorithm is proposed, and the results show that the transmission power can be significantly reduced, even with a smaller number of multiple base stations, which is of considerable interest for uplink channel communications.

In [14], an algorithm was developed to optimize the sum rate of the network under the interference constraints of primary users using beamforming and power control for each secondary user in a multiuser cognitive radio system. The interior-point method was used to solve the problem, employing a second-order cone programming (SOCP) approach.

The work described in [15] proposes an algorithm that utilizes the combination of beamforming and power control for a cognitive radio system with a base station with multiple antennas. This algorithm employs an iterative water-filling method that seeks to maximize the total rate of the secondary users, without affecting the quality of service (QoS) of the primary link, in other words, with the restriction of protecting the primary network from interferences in the cognitive radio system.

Another paper [16] considers the benefits of combining beamforming by means of multiple cells in a multiple input and multiple output (MIMO) system, where the multiple base stations can act together to optimize the corresponding beamforming in order to improve the overall performance of the system. The duality between the uplink and downlink channels is generalized for multi-cell cases using Lagrange’s theorem applied to the criterion: minimize the total transmission power subject to SINR for remote users.

The algorithm presented in [17] provides a combination of power control and beamforming in ad hoc wireless transmission networks with multiple antennas subject to constant QoS restriction. The proposed algorithm reduces the mutual interference in each node. The total transmission power of the network is minimized, while ensuring constant SINR in each receiver. Comparison was made of the performance of cooperative (COPMA) and non-cooperative (NPMG) iterative algorithms. In the case of the COPMA algorithm, users update their beamforming vectors in order to minimize the total transmission power in the network. In the case of NPMG, all the beamforming transmission vectors are updated at the start of the iteration, followed by power control.

Artificial intelligence (AI) techniques have been widely used in problems involving beamforming and power control [18,19,20,21,22,23]. The proposal of the present work is to develop an intelligent algorithm that utilizes reinforcement learning (RL) to establish an optimum policy for combination of the two techniques, beamforming (BF) and power control (PC), in sensor arrays, benefiting from the individual characteristics of each technique in accordance with SINR threshold. In this case, the great challenge of RL is to select the action (BF or PC) that, based on the learning state (SINR), is most suitable for the system. The RL algorithm used was Q-learning with an ε-greedy policy, trained using the offline method. In this case, acquirement of the most suitable techniques for indication of the action to select during each state of learning is obtained by reinforcement, employing a structure of adaptive parameters on which the algorithm operates.

It is important to emphasize that the technique proposed in this paper is not limited to use of the LMS (least mean square) procedure for fitting of the beamforming parameters. Other AI methodologies such as fuzzy logic and artificial neural networks, amongst others, can be used to improve the performance of the parameter fitting [24,25,26].

Reinforcement learning is based on the capacity of the agent to be trained to obtain knowledge while interacting with the unknown environment in which it is inserted. One of the great advantages of reinforcement training is precisely the capacity of the agent, while interacting with the unknown environment, to evolve by means of identification of the characteristics of this environment, dispensing of any need for the teacher present in supervised learning [27]. Thus, the main contributions of this work are:

- Reinforcement learning ensures that the optimal policy is found, with the most suitable technique being executed in preference to others.
- Independence in execution between beamforming and power control-these techniques are not performed sequentially, as in the above work, but alternately.
- Reduced complexity of the method, with fewer operations required, because one technique is not followed by another.
- The RL methodology proposed here is independent of the beamforming parameter fitting technique and the power control algorithm.

The paper is structured as follows: Section 2 provides a basic description of the functioning of the adaptive sensor arrays, together with a model of the input and output signals of the array, and a discussion of resolution of the beamforming problem using the LMS and power control; Section 3 presents the Q-learning algorithm, based on RL, which is the main focus of this work; Section 4 presents a proposal for an intelligent agent, employing RL; Section 5 shows the results obtained for simulations involving the agent and the sensor array; Section 6 provides the main conclusions of the work.

## 2. Adaptive Sensor Arrays

**Figure 1:**
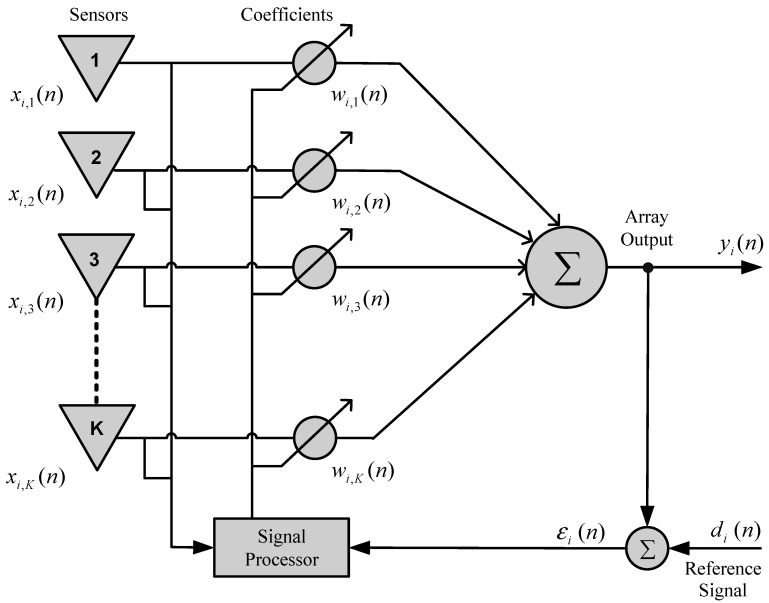
Functional diagram of an adaptive array.

Figure 1 shows a functional diagram of an adaptive sensor linear array with *K* elements. Each *k*-th element of the antenna array is spacing d should generally be equal to λ/2 (λ is the wavelength). The signal $x_{i,k}\left(n\right)$ received by the *k*-th antenna element is given by:

(1) $$x_{i,k}\left(n\right)=\overset{M}{\underset{i=1}{\sum }}\rho_{i}v_{i}\left(n\right)e^{-j\left(k-1\right)\left(\frac{2\pi }{\lambda }\right)d\cos\left(\theta_{i}\right)}+r_{k}\left(n\right)$$

where $v_{i}\left(n\right)$, $\rho_{i}$, and $\theta_{i}$ are the signal, the angle of arrival (AOA) and the attenuation of the *i*-th source, respectively. All sources have the same wavelength and $r_{k}\left(n\right)$ is the noise associated with each antenna element. The signal, $v_{i}\left(n\right)$, is modeled as a narrowband signal.

In many cases, overall reception performance is measured effectively using the SINR in the output of the array. The SINR estimates the ratio between the signal of interest with a view to noise plus interference, providing a measure of the quality of communication.

In an array of sensors, the adaptive process is conducted by means of adjusting the coefficients (weights) associated with each of the elements of the array. This adjustment is performed using a signal processor and considers a performance criterion established for the system, which could be the SINR, the mean square error, the bit error rate (BER), or any other parameter [28,29,30].

The desired characteristics of irradiation/reception, as well as the spatial filtering in an sensor array, are configured by convenient manipulation of certain array parameters, such as the number of elements (sensors), the geometry (spatial arrangement and spacing between the elements), types of antennas, and the coefficients (weights) used to adjust the signal amplitude and phase in each element.

In the system shown in Figure 1, the array output signal for the *i*-th user is given by:

(2) $$y_{i}\left(n\right)=w_{i,K}^{H}(n)x(n)$$

where $w_{i,K}^{H}(n)$ is the vector of weights for the i-th user of the system, and the index H represents the Hermitian conjugate transpose. The array output signal $y_{i}\left(n\right)$ is compared to the desired response $d_{i}\left(n\right)$, the difference between them is called the estimation error, as illustrated in Figure 1. As presented in [28], the reference signal (or desired signal) is a training sequence understood by the sensor array, sent periodically by the sources.

### 2.1. Beamforming

The objective of beamforming is to adjust the weight vectors in order to obtain the maximum SINR in the output of the array. This can be achieved by minimizing the total interference in the array output, while maintaining a constant gain for the signal of interest [5,29,30]. Taking the array output given in Equation (2), the average total output power (in W) for the *i*-th user can be written as:

(3) $$P_{i}=E[y_{i}{\left(n\right)}^{2}]=E[w_{i,K}^{H}(n)x\left(n\right)x^{H}\left(n\right)w_{i,K}(n)]$$

where *E* is the mean operator. Defining:

(4) $$R=E\left[x\left(n\right)x{\left(n\right)}^{H}\right]$$

as the autocorrelation matrix of the input signal, the average total output power of the array is given by:

(5) $$P_{i}=w_{i,K}^{H}\left(n\right)Rw_{i,K}\left(n\right)$$

The weight vectors that maximize the SINR of the array output can then be found using the following minimization problem:

(6) $$\underset{(w_{i,1},\ldots ,\;w_{i,K},\;P_{1},\;\ldots ,\;P_{k)}}{\min}\left(\overset{K}{\underset{i=1}{\sum }}P_{i}\right)\;\text{subject to }SINR_{i}\geq \delta_{i}$$

where ${\text{δ}}_{\text{i}}$, ${\text{P}}_{\text{i}}$, and $w_{\text{i},\text{K}}$ are, respectively, the smallest allowed SINR value in dB (the pre-established threshold level), the transmission power, and the beamforming vector for the *i*-th user. The aim is to obtain an optimum pair of the weight and transmission power vectors, with minimization of the total transmission power, maintaining the SINR above a pre-established threshold (${\text{δ}}_{\text{i}}$).

### 2.2. LMS Algorithm

The LMS algorithm is a method based on gradient search techniques, applied to mean square error functions, employing optimum solution of the Wiener-Hopf equation. The algorithm is based on the steepest descent method [31], in which changes in the weight vectors are made along the contrary direction of the estimated gradient vector. This can be described by:

(7) $$w_{i,K}\left(n+1\right)=w_{i,K}\left(n\right)-\mu \hat{\nabla }(n)$$

where μ is a scaling constant that controls the rate of convergence and stability of the algorithm (adaptation step), and $\hat{\nabla }\left(n\right)\;$ is the vector gradient estimated from the quadratic error in relation to $w_{i,K}\left(n\right)$.

The error is obtained between the output of the filter, ($y_{i}\left(n\right)=w_{i,K}^{H}\left(n\right)x(n)$), and the reference signal, $d_{i}\left(n\right)$, so that:

(8) $$\varepsilon_{i}\left(n\right)=d_{i}\left(n\right)-\;w_{i,K}^{H}\left(n\right)x\left(n\right)$$

The stop criterion or convergence criterion of the weights using LMS algorithm is the variation of the mean square error at each iterarion, *i.e.*:

(9) $$\left|{\varepsilon_{i}}^{2}\left(n\right)-{\varepsilon_{i}}^{2}\left(n-1\right)\right|<\xi$$

where ξ ia threshold defined by the designer based on their application that limits the number of iterations.

### 2.3. Power Control

An important benefit derived from beamforming and consequent increase in the SINR is the possibility of reducing the signal transmission power. This improves the energy efficiency of the system and reduces interference between the users, ensuring that each signal is transmitted with the lowest power required to maintain a good quality connection.

The control of power in sensor arrays is based on a selected quality criterion, which in the present case was the SINR. During this process, the power is reduced in order to satisfy the restrictions shown in Equation (6). Based on papers presented at [8,9,10,11], the updating of the power is given by:

(10) $$P_{i}\left(n+1\right)=P_{i}\left(n\right)\frac{\delta_{i}}{SINR_{i}}$$

where it can be seen that convergence is achieved when $SINR_{i}=\delta_{i}$. In the calculation of Equation (10), it is necessary to know the value of the $SINR_{i}$ in the moving terminal, which can be estimated from the minimum mean square error of the beamforming step (LMS), as described by:

(11) $$SINR_{i}=\frac{1-\min E[{\varepsilon_{i}}^{2}\left(n\right)]}{\min E[{\varepsilon_{i}}^{2}\left(n\right)]}$$

where $SINR_{i}$ represents the estimated SINR for each user.

Rearranging Equation (10) as a function of the minimum mean square error gives the final expression for calculation of the power:

(12) $$P_{i}(n+1)=\delta_{i}P_{i}(n)\frac{\min E[{\varepsilon_{i}}^{2}\left(n\right)]}{1-\min E[{\varepsilon_{i}}^{2}\left(n\right)]}$$

## 3. Reinforcement Learning

Reinforcement learning is a technique whereby an apprentice agent attempts to maximize a performance parameter based on the reinforcement it receives while interacting with an unknown environment. Its use is recommended when there are no a priori models available, or when it is not possible to obtain appropriate examples of situations to which the apprentice agent will be exposed. The agent that lacks previous knowledge learns by means of interaction with the environment, being rewarded for its actions and thereby discovering the optimum policy [32].

In a reinforcement learning system, the state of the environment is represented by a set of variables, known as the state space, which are perceived by the senses of the agent. An action chosen by the agent changes the state of the environment, and the value of this transition of states is passed to the environment by means of a scalar reinforcement signal (reward signal). The objective of the technique is to lead the agent to selection of the sequence of actions that would tend to increase the sum of the reward signal values.

**Figure 2:**
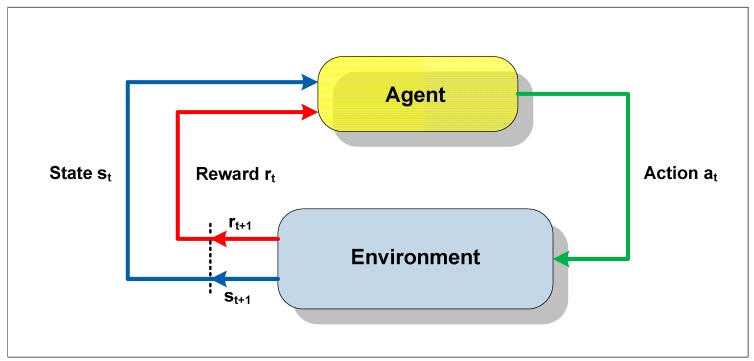
Scheme of interaction between the agent and environment.

The agent moves autonomously in the state space, interacting with the environment and learning about it through experimentation. Each time that the agent performs an action, an external training entity (critic), or even the environment, can give it a reward or a penalty, indicating how desirable it would be to reach the resulting state [33]. Hence, the reinforcement does not always signify an advance, as it can also inhibit the agent in relation to the action executed. Figure 2 provides a generic scheme of the notion of learning by reinforcement.

The goal of the RL method is to guide the agent towards taking actions that would result in maximizing (or minimizing) the sum of the reinforcement signals (numerical reward or punishment) received over the course of time, known as the expected return, which does not always signify maximizing the immediate reinforcement to be received [34].

The expression describing the sum of the reinforcement signals in an infinite horizon is given by:

(13) $$R_{t}=r_{t+1}+\gamma .\;r_{t+2}+\ldots =r_{t+1}+\;\overset{\infty }{\underset{k=1}{\sum }}\gamma^{k}.r_{t+k+1}$$

where $R_{t}$ represents the return (sum of the reinforcements) received over the course of time, $r_{t+k}$ is the immediate reinforcement signal, and γ is the discount factor, defined in the interval 0 ≤ γ ≤ 1, that ensures that $R_{t}$ is finite. If γ = 0, the agent has a myopic view of the reinforcements, maximizing only the immediate reinforcements. If γ = 1, the reinforcement view covers all future states giving the same importance to gains at the moment and any future gain.

The behavior that the agent should adopt in order to achieve maximization (or minimization) of the return is known as the policy and can be expressed by π. According to [34], a policy *π (s,a)* is a mapping of states (*s*) in actions (*a*) taken in that state, and represents the probability of selecting each one of the possible actions, in such a way that the best actions correspond to the greatest probabilities of selection. When this mapping maximizes the sum of the rewards, the optimum policy has been achieved.

Evaluation of the quality of the actions taken by the agent involves application of the concept of state-action value function, *Q(s,a)*, which is a value that provides an estimate of how good it is for the agent to be in a given state (*s*) and take a given action (*a*), when it is following any policy π. The term *Q(s,a)* represents the expected value of the total return for the state $s_{t}=s$ (the present state), which is the sum of the reinforcements, taking into account the rate of discount (γ), as described in expression:

(14) $$Q^{\pi }(s,a)=E_{\pi }\left\{\overset{\infty }{\underset{k=0}{\sum }}\gamma^{k}r_{t+k+1}|s_{t}=s,\;a_{t}=a\right\}$$

Two central questions in relation to reinforcement learning are presented in [32]:

- Given a policy *π(s,a)*, what is the best way to estimate *Q(s,a)*?
- Given an affirmative response to the preceding question, how can this policy be modified so that *Q(s,a)* approaches the optimum value of this function, and how can the consequent corresponding optimum policy be obtained?

The literature suggests various algorithms that can be used in replying to these questions. However, in the present work it was decided to use the Q-learning algorithm developed by [35], which offers advantages including the fact that it can directly approach the optimum value of *Q(s, a)*, irrespective of the policy employed. The values of *Q(s, a)* are updated according to:

(15) $$Q\left(s,a\right)=Q\left(s,a\right)+\alpha \left[r_{t+1}+\;\gamma .max_{a}Q\left(s_{t+1},\;a\right)-Q\left(s,a\right)\right]$$

where α is the learning rate (0≤α<1) and γ is the discount rate *(0 ≤ γ < 1)*.

The Q-learning algorithm is presented in Algorithm 1. The episode mentioned in this algorithm is characterized by a sequence of states ending in a final state.

**Algorithm 1.** Q-learning algorithm.1: Initialize *Q (s, a)* randomly;2: **Repeat** (for each episode)3: Initialize *s*;4: **Repeat**5: Choose *a* for *s* using the policy π;6: Given the action *a*, watch *r*, *s’*;7: $Q\left(s,a\right)=Q\left(s,a\right)+\;\alpha \left[r_{t+1}+\gamma .\;max_{a}Q\left(s_{t+1},\;a\right)-Q\left(s,a\right)\right]$8: *s*

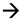

*s’*9: **until** the final state is reached;10: **until** the number of episodes is reached

Given that convergence of the algorithm can only be guaranteed if all the state-action pairs are visited an infinite number of times, selection of the policy to be used in the Q-learning algorithm must ensure that all the pairs have non-null probability of being visited. This can be achieved using an ε-greedy policy, defined by:

(16) $$\pi \left(s,a\right)=\{\begin{array}{c} \begin{array}{c} 1-\varepsilon +\frac{\varepsilon }{\left|A\left(s\right)\right|},\;if\;a=\;a^{*}=\arg\underset{a}{\max}Q\left(s,a\right) \end{array}\\ \frac{\varepsilon }{\left|A\left(s\right)\right|},\;a\neq a^{*} \end{array}$$

This policy consists in a choice of the associated action to the highest value of Q with $1-\varepsilon +\frac{\varepsilon }{\left|A\left(s\right)\right|}$ probability and random selection of any other action with $\frac{\varepsilon }{\left|A\left(s\right)\right|}$ probability, where *|A(s)|* is the number of possible actions to be executed from *s*, and ε is the control parameter between greed and randomness.

## 4. Proposed Solution (Intelligent Agent Project)

In this problem, the aim of the reinforcement learning modeling is to find an optimum policy able to indicate the most suitable techniques that the agent should select among the actions available, considering beamforming and power control.

From the point of view of reinforcement learning, the problem can be modeled as follows: the state of the environment is represented by a discrete set of SINR values, so that S = {SINR_1_, SINR_2_, …, SINR_m_}, where SINR_m_ represents the maximum value defined for the SINR.

In each state *s* ∈ *S*, the deciding agent must select an action *a* from a set of actions available in the state *s*, denoted by *A(s)*. The possible actions available for each state are beamforming (BF) and power control (PC). The Q-learning algorithm governs the decision to explore or take advantage, using the policy known as *ε*-greedy. This policy is defined in the algorithm for selection of the action that possesses the highest value for the utility of the state (greedy criterion), with probability (1 *− ε*), and for random action, with probability *ε*.

As a consequence of selection of an action *a* ∈ *A(s)*, starting from state *s* and at instant of decision *t*, the deciding agent receives a reward *r_t_(s,a)*. The selection of action *a* alters the perception of the agent in relation to the environment, leading to a new state *s_t+1_* that conducts it to a new instant of decision *t + 1*. When the reward is positive, it is seen as a profit or prize, and when it is negative, it is seen as a cost or punishment. In definition of the return function (reward), an indication should be provided of the objective to be achieved by the algorithm. In this problem, an attempt is made to minimize the total transmission power, maintaining constant the gain of the desired signal, and keeping the SINR above a pre-established threshold δ. The reward was therefore defined as being positive when the SINR approached the threshold, and negative when the opposite was true.

Once the states, actions, and return function had been defined, the next step was the process of training the agent, as shown in Algorithm 2. At the start of the learning process, the agent has no knowledge of the result obtained by choosing a particular action, so it performs various actions and observes the results. For a while, the agent explores many actions that result in increasingly greater rewards, and gradually tends to repeat them (exploration); after this, it acquires knowledge from the actions, and can sometimes learn to repeat those that result in greater rewards (exploitation).

Algorithm 2 requires information for the parameters (line 1): learning coefficient (α), parameter for regulation of the greedy criterion (ε), discount rate (γ), and LMS stop criterion (ξ). Subsequently, the matrix *Q* is initiated using random values (line 2), and the initial state is determined randomly within the values defined in *s* (line 4). An available action is chosen for *s* (line 6), and according to the action selected, the corresponding technique (BF or PC) is executed. If the action selected is BF, the algorithm will adjust the weights while the determined condition (line 8) remains satisfied, and if not, will update the transmission power in accordance with the equation presented in Algorithm 2 (line 16).

Assuming that the initial action PC is chosen, a random value was given for the *min_mse* variable equal to 2 (which is the minimum value of the error) and then the powers were updated. Otherwise, *i.e*., if the chosen initial action is BF, the *min_mse* used to update the powers is based on the last iteration of the error vector $\varepsilon_{i}$ in BF operation. The power is a vector of positions, where *M* is the number of signals that are addressing the array of sensors. In executing the available actions for *s*, the reward (*r*) (line 19) and a new state $\left({\text{ s}}^{\text{'}}\right)$ are reached, according to the equation in line 20, and the matrix Q is updated (line 21). The process (lines 6–22) repeats itself until a final state is found, which in this work was defined by the SINR threshold. After attaining the final state, the algorithm is executed (from line 4) until the defined number of repetitions is completed. The stages of the Q-learning algorithm applied to the sensor array in the agent training step are shown in Algorithm 2.

At the end of the training, a *Q* matrix is constructed, which is utilized in the functioning of the agent. It is important to note that for each BF action, there is storage of a matrix ***W***(*s*, 1), containing all the optimum weight values, together with a matrix ***P***(*s*, 2), for each PC action, corresponding to all the transmission power updates. Whenever the environment is changed, a new execution of Algorithm 2 is required.

**Algorithm 2.** Sensor array Q-learning algorithm.1: **Require:** (α, ε, γ,  ξ, M)2: Initialize *Q (s*,* a)*3: **While** maximum episodes are not reached **Do**4:  Initialize *s*        *% initial state*5:  **While** final state is not reached **Do**6:   Choose *a* according to *ε*-greedy rule; *% a =1(BF) or a=2 (PC)*7:   **If** a==1      % Beamforming8:    **While**
$\left|{\varepsilon_{i}}^{2}\left(n\right)-{\varepsilon_{i}}^{2}\left(n-1\right)\right|<\xi$
**Do**  $\%\;threshold\;less\;than\;10^{-4}$9:     **For** i=1, 2, …, M10:       $w_{i}\left(n+1\right)=w_{i}\left(n\right)+\mu x(n)\varepsilon_{i}(n)$11:     **End-For**12:    **End-While**13:   **End - If**14:   **If** a==2     *% Power Control*15:     **For** i=1, 2, …, *M*16:       $P_{i}=\delta P_{i}\frac{min\_mse}{1-min\_mse}$
*% Updated powers*17:     **End-For**18:   **End - If**19:   Watch *r % Reward*$20:\;\;s^{'}=(1-min\_mse)/min\_mse$     *% New State*21:$\;Q\left(s,a\right)=Q\left(s,a\right)+\;\alpha \left[r_{t+1}+\gamma .\;max_{a}Q\left(s_{t+1},\;a\right)-Q\left(s,a\right)\right]\;$
*% Updating Q-table*22:   *s*

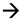

*s’*23:   **End-While**24: **End-While**25: **return**
*Q(s,a) % Q-values Matrix*

Algorithm 3 shows the functioning of the agent, where *j* is the processing cycle, corresponding to selection of an initial state, execution of an action, and attainment of a new state.

**Algorithm 3.** Algorithm for functioning of the agent.2: **If** (environment changes)3: training *% Algorithm 2*4: **Else**5:  Choose an initial state *s*6:  **For** j** from** 1** until** max_value** Do**7: Choose *a_max_* for *s* and run8:   *s*

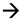

*s’*9:  ***End-For***

## 5. Simulation and Results

The functioning of the algorithm was demonstrated for two sources (*M* = 2) using simulation of a situation with angles of 90° and 30° for the desired and interferent signals, respectively. The target SINR was 2 dB, and the initial powers were set at 1 W for both sources. All sources were modeled with polar binary signals ($v_{i}\left(n\right)\;\in \;\left\{-1,1\right\}$) random uniform distribution. The noise at each antenna element was modeled as Additive white Gaussian noise (AWGN) with variance $\sigma^{2}$. The parameters used in the simulation are listed in Table 1.

**Table 1:** Simulation Parameters.

Parameters	Value
Number of elements in the array (*K*)	8
Number of signals (*M*)	2
Initial transmit power (P_0_)	1 W
SINR threshold (δ)	2 dB
Step Adaptation (μ )	0.001
Noise Variance $\left(\;\sigma^{2}\;\right)$	0.1
Distance between each element of the array (*d*)	λ/2
Learning Rate ( α )	0.1
Discount Factor ( γ )	0.9
Greedy Rule ( ε)	0.2
Attenuation of the *i*-th source (${\text{ρ}}_{\text{i}}$)	1

In this paper, a linear array of sensors with eight elements and two signal sources was used for a desired source and an interfering source. A linear array with *K* elements can create up to *K* − 1 nulls in the direction of the interfering source. When the number of unwanted (interfering) sources is close to such a limit, the attenuation of unwanted signals is reduced and there are excess gains (greater than the gain attributed to the desired signal) in the proximity of the desired and undesired angles. Thus, the use of two sources is in the range between the limits established for the performance of the system.

The distance between the array elements (*d*) is limited by the value λ/2. This limitation avoids the production and overlapping of side lobes. The unit transmission powers were used to initialize the power control algorithm and to facilitate the calculations. The choice of μ in the adaptation step is experimentally determined in order to provide stability of the algorithm. The higher the adaptation step value, the higher the convergence speed. However, the excess error also becomes larger, which is undesirable. The  α, γ, and ε values were obtained after several simulations.

The states of the environment were represented by discrete SINR values between −0.8 and 5, which corresponded to index values in the range 1–18. Each action was identified with the label 1 or 2, indicative of beamforming and power control, respectively. An ε-greedy policy was adopted, with 80% possibility of selecting the better action.

Two simulations were performed using the parameters shown in Table 1, but with the variation of the noise (σ^2^) changed to 0.3 in the second simulation. In each simulation, the training was configured to execute 10, 50 and 250 episodes.

The results of the first simulation are shown in Table 2, indicating the policies obtained after each different episode. Each line corresponds to a SINR value, and the columns correspond to the beamforming (BF) or power control (PC) processes.

**Table 2:** Policy Improvement of the agent.

		Policy 10		Policy 50		Policy 250	
INDEX	SINR(dB)	BF	PC	BF	PC	BF	PC
1	−0.8	0.5	0.5	1	0	0	1
2	−0.6	0.5	0.5	1	0	1	0
3	−0.4	0.5	0.5	1	0	0	1
4	−0.2	0	1	1	0	1	0
5	0	0	1	0	1	0	1
6	0.2	0	1	0	1	0	1
7	0.4	0	1	0	1	0	1
8	0.6	0	1	0	1	0	1
9	0.8	1	0	1	0	0	1
10	1	0	1	0	1	0	1
11	1.2	0	1	1	0	0	1
12	1.4	0	1	0	1	0	1
13	1.6	0	1	0	1	0	1
14	1.8	0	1	0	1	0	1
15	2	**Destination**		**Destination**		**Destination**	
16	3	1	0	0	1	1	0
17	4	1	0	1	0	1	0
18	5	1	0	1	0	1	0

The values given in Table 2 correspond to the probability of selecting each technique, for the range of discretized SINR values. The optimum policy was obtained after 250 episodes, and was adopted for testing the agent.

**Figure 3:**
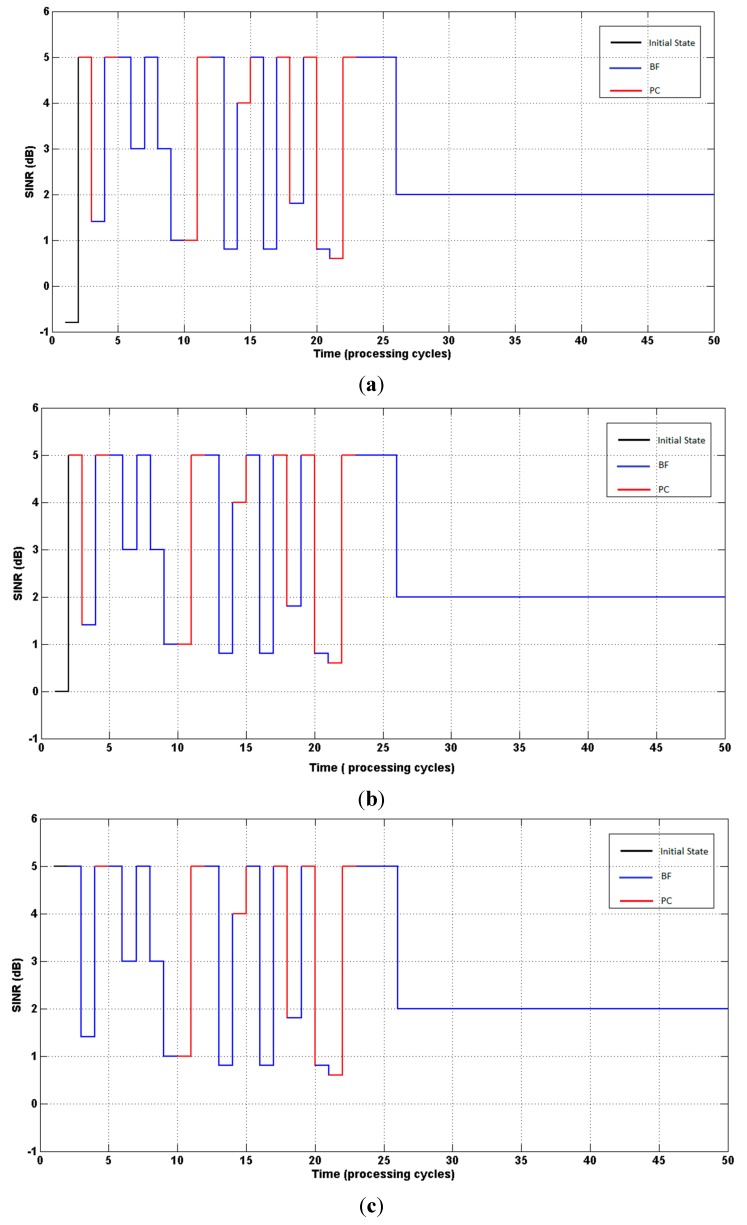
(**a**) System response. Agent started with SINR = −0.8 dB; (**b**) System response. Agent started with SINR = 0 dB; (**c**) System response. Agent started with SINR = 5 dB.

Figure 3 present (on the ordinate axis) the states (SINR), and the curves indicate the evolution of the SINR until reaching the target value (δ = 2).

**Figure 4:**
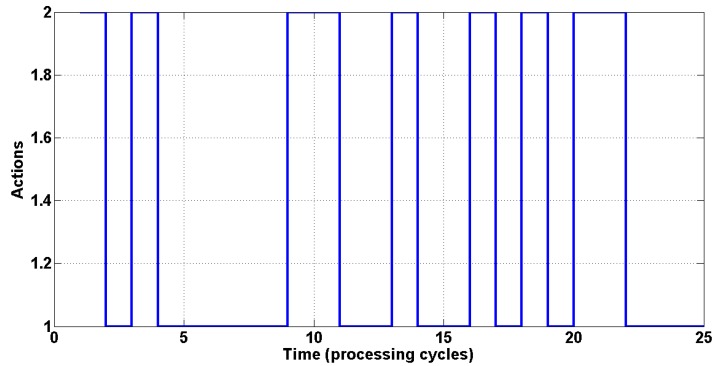
The switching sequence among the two techniques.

The switching sequence using an initial SINR of −0.8 is illustrated in Figure 4, with the actions executed (beamforming or power control) indicated on the ordinate axis, and the curve showing the order of execution in each processing cycle. Index 1 indicates execution of beamforming, and Index 2 indicates power control.

Table 3 presents the simulation results obtained using the same parameters, but with variance of the noise equal to 0.3.

**Table 3:** Policy Improvement of the agent.

		Policy 10		Policy 50			Policy 250	
INDEX	SINR(dB)	BF	PC	BF	PC	BF		PC
1	−0.8	0.5	0.5	0	1	0		1
2	−0.6	0.5	0.5	0	1	0		1
3	−0.4	0.5	0.5	1	0	0		1
4	−0.2	0	1	1	0	0		1
5	0	0.5	0.5	0.5	0.5	0		1
6	0.2	0	1	0	1	0		1
7	0.4	0	1	0	1	0		1
8	0.6	0	1	0	1	0		1
9	0.8	1	0	0	1	0		1
10	1	0	1	0	1	0		1
11	1.2	0.5	0.5	0	1	0		1
12	1.4	0	1	0	1	1		0
13	1.6	0	1	0	1	0		1
14	1.8	0	1	0	1	0		1
15	2	**Destination**		**Destination**		**Destination**		
16	3	0	1	1	0	1		0
17	4	0	1	1	0	1		0
18	5	1	0	1	0	1		0

**Figure 5:**
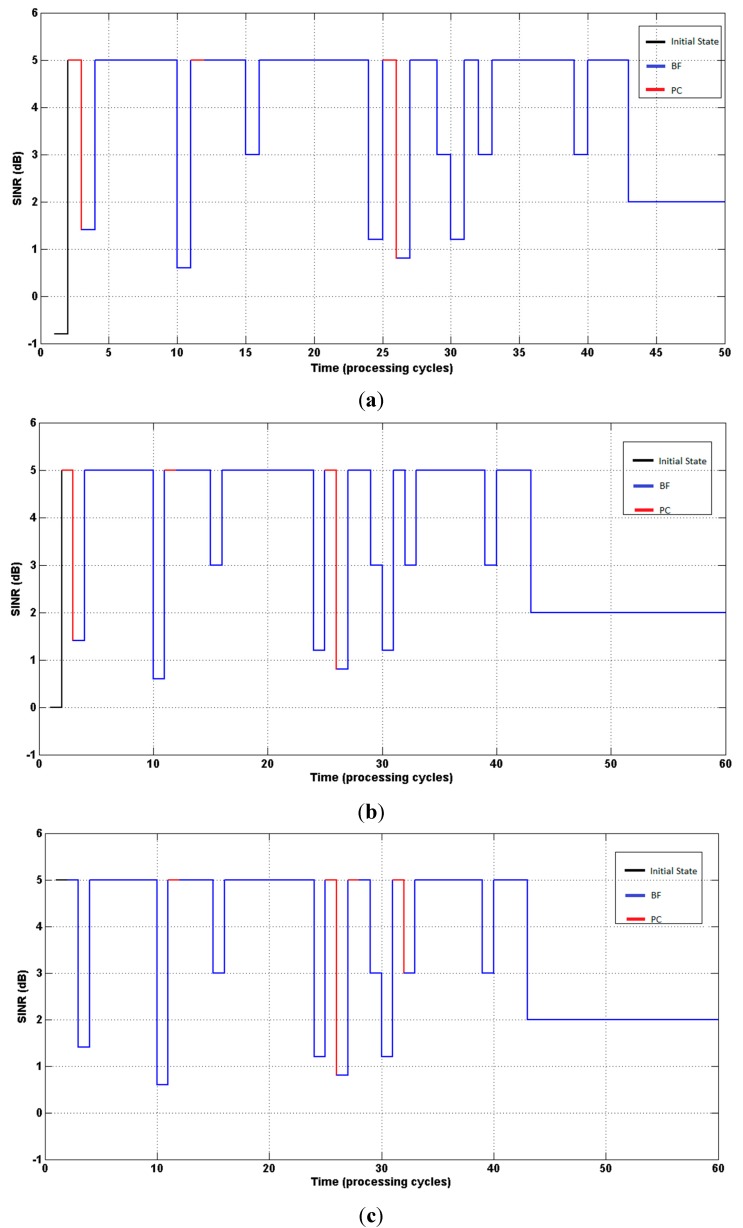
(**a**) System response. Agent started with SINR = −0.8 dB; (**b**) System response. Agent started with SINR = 0 dB; (**c**) System response. Agent started with SINR = 5 dB.

From Table 2 and Table 3, it can be seen that power control was selected in more states, compared to beamforming, which demonstrates the independence of the algorithm in selecting the optimum policy, in contrast to other procedures [8,9,10,17] in which only the power is updated once beamforming convergence is reached. Figure 5 illustrate the evolution of the SINR until the target value is reached (second simulation).

**Figure 6:**
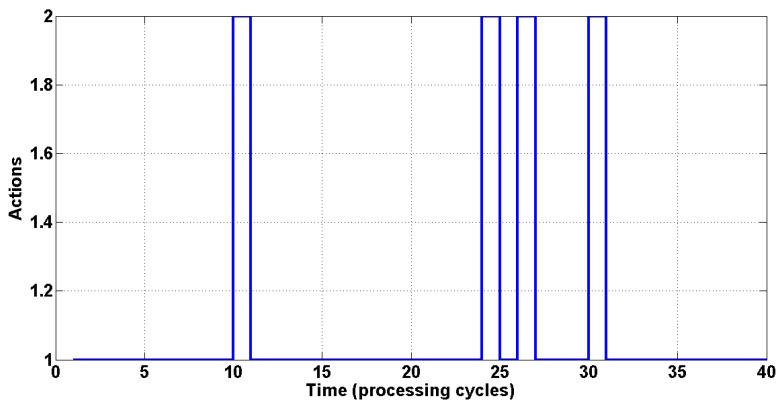
The switching sequence among the two techniques.

Figure 6 presents the switching sequence for an initial SINR of 5, where the ordinate axis shows the actions executed (beamforming or power control) and the curve indicates the order of execution in each processing cycle. Index 1 indicates the execution of beamforming and Index 2 indicates the execution of power control.

It can be seen that even with increase of the variance of the noise from 0.1 to 0.3, the proposed algorithm was effective in selecting the optimum policy, requiring only one new training, given that the environment was modified. This is an important point, and shows the robustness of the algorithm when faced with new noise conditions.

## 6. Conclusions

The use of beamforming and power control individually in sensor arrays has its benefits, but it can be seen that by employing them jointly there is an increase in system performance. Several studies have used beamforming and power control jointly, but this paper presents a new control method employing a combination of beamforming and power control. The algorithm presented uses the technique of reinforcement learning to obtain the optimum policy for selection between beamforming and power control in sensor arrays.

It can also be seen that the proposed technique reduces the computational cost, as the techniques are selected independently. For example (Table 2), using the optimum policy obtained after 250 episodes, it was found that in many cases it was not necessary to execute the LMS algorithm. This resulted in lower computational cost and reduced complexity of the proposed method.

From the simulations performed, it could be concluded that reinforcement learning offers an effective way of implementing a policy of switching between beamforming and power control in sensor arrays, benefiting from the advantages of both techniques.
